# The Effect of Loneliness on Cognitive Functioning Among Healthy Individuals in Mid- and Late-Adulthood: Evidence From the Canadian Longitudinal Study on Aging (CLSA)

**DOI:** 10.3389/fpsyg.2021.701305

**Published:** 2021-09-03

**Authors:** Aki-Juhani Kyröläinen, Victor Kuperman

**Affiliations:** ^1^Reading Lab, Department of Linguistics and Languages, McMaster University, Hamilton, ON, Canada; ^2^Department of Applied Linguistics, Brock University, St. Catharines, ON, Canada

**Keywords:** aging, cognitive functioning, loneliness, memory, CLSA

## Abstract

There is a consensus that loneliness correlates with an increased risk of cognitive impairment and rapid cognitive decline. However, it has yet to be determined how loneliness influences cognitively healthy aging. This study makes use of the large, nationally representative Canadian Longitudinal Study of Aging (CLSA) to address this question. Based on the baseline and first follow-up datasets collected 3 years apart (*n* > 20,000 healthy individuals), we found that higher perceived loneliness predicted decreased scores in the immediate recall test at baseline and in two tests of prospective memory at first follow-up 3 years after baseline. We also examined whether a single-item measurement of loneliness widely used in the field of gerontology, including CLSA, has predictive validity, i.e., can contribute to the prognosis of a future level of cognitive functioning. We found low predictive validity and low test-retest (baseline to follow-up) reliability of this measurement type. These findings impose constraints on proposed accounts of loneliness as a risk factor and methods of examining its relation to cognitive aging.

## 1. Introduction

Maintaining satisfactory cognitive functioning is a critical part of healthy aging. One of the oft-proposed clinically significant risk factors for cognitive abilities in the aging population is loneliness (de Jong-Gierveld and Havens, 2004; Heinrich and Gullone, 2006; Menec et al., 2019). Loneliness is defined as a subjective feeling of dissatisfaction with the extent or intensity of one's social interactions relative to one's social desires (Shankar et al., 2013; Lara et al., 2019). Several longitudinal studies of aging (Shankar et al., 2013; Boss et al., 2015; Kuiper et al., 2016; Lara et al., 2019) have indeed demonstrated that an increased risk of low cognitive functioning and rapid cognitive decline over time is associated with higher subjective levels of loneliness. During the current COVID-19 pandemic, international reports indicate both a higher incidence of perceived loneliness due to social distancing and exacerbation of the effects that loneliness has on mental health (Groarke et al., 2020; Killgore et al., 2020; Luchetti et al., 2020). These correlations led researchers to suggest routine screening or interventions targeting the unmet social needs and perceived loneliness (Dickens et al., 2011; Lara et al., 2019) in mature adults.

Despite the apparent consensus on the existence of the loneliness effect, it is presently unclear how strong the effect of loneliness is and what facets of cognitive abilities it influences. For instance, a recent meta-analysis (Kuiper et al., 2016) reports very high heterogeneity (*I*^2^ = 0.88, *p* < 0.01) in the presence and magnitude of observed correlations between loneliness and declining cognitive functioning in mature adults. Another systematic review (Boss et al., 2015) indicates that some of the reported harmful effects of loneliness on cognitive abilities disappear when controlling for demographic, psychological, and social risk factors. Finally, the loneliness effect is strongly modulated and in some circumstances canceled out by measures of “cognitive reserve” defined as the degree of an individual's resilience to a pathological decline in cognitive functioning determined by the lifetime accumulation of and current participation in cognitively stimulating activities (e.g., education, occupation, physical exercise, and social interactions) (Conroy et al., 2010; Evans et al., 2018; Farina et al., 2018). This suggests that while loneliness is pervasive in mature adulthood (Hawkley and Cacioppo, 2010), the magnitude and the prevalence of the impact that it has on cognitive functioning are presently undetermined.

Not only is the existing evidence on loneliness effects of cognitive aging mixed, but it is also limited relative to research on other types of social relationships. This is despite multiple demonstrations that loneliness is an independent and stronger predictor of the incidence of cognitive impairment than a related construct of social isolation (de Jong-Gierveld and Havens, 2004; Ellwardt et al., 2013; Holwerda et al., 2014). Also, with only a few exceptions, including Lara et al. (2019) and select studies reviewed in Boss et al. (2015), prior work concentrates on loneliness in the context of clinical depression or pathological cognitive deterioration, including dementia and mild cognitive impairment (e.g., Lobo et al., 2008). Yet perceived loneliness is also prevalent in healthy aging and has been argued to have a detrimental effect on individual life satisfaction and psychological and social well-being even in individuals not meeting diagnostic criteria for cognitive impairments (Heinrich and Gullone, 2006). Given the clinical, societal, and economic importance of healthy aging, research on loneliness and cognitive functioning can benefit from new, comprehensive evidence. The first goal of this paper is to provide longitudinal evidence on what cognitive abilities are affected by loneliness the most.

An additional goal of this paper is methodological. It stems from the fact that the most commonly used way of measuring the construct of perceived loneliness in gerontological research and mental state diagnostics is to ask a single-item question on how lonely an individual has felt recently (see details below). As is evident from references above, this measurement of loneliness has concurrent validity, i.e., when measured at the same time, loneliness correlates with the cognitive and psychological performance of an individual (see references above). A less explored issue is whether loneliness can also be used predictively—to predict one's future cognitive functioning and its change over time, as advocated by Shankar et al. (2013). If measurements of loneliness have this predictive validity, they can be used to forecast the likely temporal course of cognitive functioning for a given person, which is knowledge of primary importance for medical practitioners and caretakers. If not, perceived loneliness can still be used for a concurrent diagnosis of cognitive state, as is indeed done in the Geriatric Mental State test GMS-AGECAT (Copeland et al., 1986), but not for the prognosis of future cognitive impairment or decline. To our knowledge, the determination regarding predictive validity of loneliness has not yet been made, and this study addresses it using longitudinal data on mature adults.

This study examines the link between feelings of loneliness and cognitive functioning in healthy mid- and late-adulthood using data from the Canadian Longitudinal Study of Aging (CLSA) (Raina et al., 2009, 2019; Menec et al., 2019), a large, nationally representative sample of 45+ year olds. The goal of the CLSA is to provide a national longitudinal research platform covering adults from all 10 Canadian provinces while simultaneously collecting comprehensive data and biological samples. The design of the CLSA comprises two cohorts: the Tracking (21,241 individuals [unweighted]) and the Comprehensive one (30,097 [unweighted]). The individuals in the Comprehensive cohort were randomly selected from within 25–50 km of 11 data collection sites (DCSs) in seven provinces. The details of the recruitment process are described in Raina et al. (2009) and in Raina et al. (2019). In this study, we make use of the data available in the Comprehensive cohort.

The CLSA data provide access both to a larger sample than those available in earlier longitudinal studies reviewed above (by factor of 2–8 after applying exclusion criteria) and a larger selection of cognitive functioning tests as well as variables tapping into cognitive reserve (Farina et al., 2018) and other factors of relevance. Furthermore, CLSA uses census-based sampling weights that remove inaccuracies due to sampling error or an imbalanced representation of the Canadian population. In this study, we made use of CLSA Sample Weights Version 1.2. This coverage and accuracy support our first goal: to pinpoint the facets of cognitive functioning that are particularly impeded by loneliness in healthy mature and older adults over time. This determination is important for explaining the nature of the link between loneliness and aging.

To reiterate, our second goal is to establish whether perceived loneliness has predictive validity, i.e., whether the baseline level of perceived loneliness can predict cognitive performance at follow-up. As part of estimating the predictive power of loneliness for the future cognitive functioning we compute the test-retest (baseline to follow-up) reliability of the loneliness measurements in CLSA data: if this reliability is low, predictive validity is unlikely. This paper focuses on the most common of several existing operationalizations of loneliness. Specifically, the decisive majority of cross-sectional and longitudinal research on loneliness operationalizes this critical concept as a single-item question either in a yes-no format (e.g., “Do you feel lonely at the present time?”) or using a Likert scale (e.g., “How often do you experience loneliness?,” see Boss et al., 2015). As an example of how prevalent this approach is, a recent meta-analysis (Boss et al., 2015) lists ten studies examining loneliness in the aging population: seven of those made use of single-item questions to measure loneliness while the remaining three used either the 3-item, short form of the Revised-UCLA Loneliness Scale (Shankar et al., 2013) or the six-item De Jong-Gierveld Loneliness Scale (Wilson et al., 2007; Schnittger et al., 2012). Moreover, the single-item approach to measuring loneliness is adopted in the influential diagnostic test of the Geriatric Mental State GMS-AGECAT: (Copeland et al., 1986, i.e., “Do you feel lonely?” with three response options “no/mildly/severely”), see also Tan et al. (2019). The CLSA sides with the majority in this regard and also makes use of a single-item question (“How often did you feel lonely in the past week?”) to measure the construct of loneliness (see Newall and Menec, 2020, for discussion about relevant single-item indicators in CLSA in general). Even though these measurements clearly interrogate one's feelings of loneliness in the present or very recent past, several longitudinal studies use at-baseline loneliness measurements as predictors of cognitive performance at follow-ups (see Wilson et al., 2007; Shankar et al., 2013; Holwerda et al., 2014, for relevant proposals or analyses). Thus, we expect our examination of predictive validity and test-retest reliability in longitudinal CLSA data to shed light on potential methodological constraints associated with the widely accepted use of the single-item measurement of loneliness in the field of gerontology.

## 2. Method

### 2.1. Participants

We used data from the Comprehensive cohort of 30,097 participants (unweighted) in the Canadian Longitudinal Study of Aging (CLSA). This cohort (age range 45–86 y.o., *Mdn* = 62 years; 51% female at baseline) used the Canadian national census as a sampling frame to create a representative sample of middle-age and mature Canadian adults. The following exclusion criteria were used: inability to complete the survey either in English or French; cognitive impairment at the time of contact; resident of the three territories; full-time member of the Canadian Armed Forces; resident in a long-term care institution; and living on Federal First Nations reserves or other First Nations settlements. All CLSA participants provided written consent before participating in the study.

The participants are to be observed at regular 3-year intervals for 20 years. Beyond the baseline data, this study includes data from the first available follow-up of the Comprehensive cohort that was administered 3 years after baseline and obtained within-participant data from nearly 28,000 individuals (unweighted).

Since our focus is on healthy aging, we removed from consideration those participants who screened positively on the CESD-10 depression scale (Radloff, 1977) or who were reported to have received mental or medical professional care during the 12 months prior to baseline testing. Because language background may affect performance in the language-related cognitive tests, we also restricted our sample to individuals born in Canada and speaking an official language of Canada as their first language. This led to a data pool of 20,355 individuals at baseline (unweighted). We further removed data points with missing or invalid responses for cognitive test scores or control variables (defined below) at either baseline or follow-up. The resulting pool with complete baseline and follow-up data contained 12,320 individuals (unweighted) ranging in age from 45 to 86 (*Mdn* = 60; 48% female).

### 2.2. Materials and Procedure

At both baseline and follow-up, all participants completed a highly consistent set of questionnaires, psychological tests, and physical assessments. This study focuses on select measurements of psychological functioning, social functioning, lifestyle, and socio-demographic context and their change over time.

#### 2.2.1. Dependent Variables

Psychological tests of memory and executive function provided multiple dependent variables for this study. Our description below follows Tuokko et al. (2017): also, we refer readers to Tuokko et al. (2017) for the rationale for selecting cognitive measures and details on administration procedure and test reliability. The sole memory test in the battery, the Rey Auditory Verbal Learning Test (RAVLT; Rey, 1964) taps into both learning and retention. The test presents participants with a 15-item word learning list read at the rate of one per second, and records the words in the order in which the participant says them, immediately after learning (*REY1* score) and after a 5-min delay (*REY2* score). The score is the number of correctly named words, regardless of the order.

The tests of executive function included the Mental Alternation Test of mental flexibility and processing speed (Teng, 1995). The test requires a participant to alternate between the numbers 1–26 and the letters of the alphabet (i.e., 1-A, 2-B, 3-C, etc.). The score (*MAT*) is the number of correct alternations produced within 30 s. Fluency was measured via two tests. The Animal Fluency task (Read, 1987) asked participants to name as many animals as possible in 60 s. Its scores (*AFT1* and *AFT2*) were the number of different existing animals recited while including or excluding breed and scientific taxonomic sub-species, respectively. The Controlled Oral Word Association Test (Lezak et al., 2004) tapped into phonological fluency and knowledge and consisted of three one-minute trials asking participants to name as many words as possible that begin with the letter A, F, or S, respectively. The summed number of correctly produced words is the task score (*FAS*).

An additional test was the Prospective Memory Task (Loewenstein and Acevedo, 2004), which measures the ability to remember to perform a planned action at or by a particular time or in response to a known event. The CLSA battery contains both event-based and time-based prospective memory tasks using cues after either 15- or 30-min delays. The scoring system is based on three criteria: intention to perform, accuracy of response, and need for reminders. Scores for the event-based (*PMT*) and time-based (*TMT*) tasks, respectively, are summed across the criteria. A final cognitive test we consider is the Stroop Test (Troyer et al., 2006; Bayard et al., 2009, 2011; Moroni and Bayard, 2009). It taps into inhibition, attention, mental speed, and mental control, and involves (i) naming the ink color of the dots printed on the card; (ii) naming the ink color of non-color words printed on the card; and (iii) naming the ink color of the color words, without reading aloud those words. The score (*STP*) is the difference between (iii) and (i). Cognitive test scores were obtained both at baseline and follow-up (see Table 1) and are the dependent variables of this study.

**Table 1:** Descriptive statistics for cognitive tests and *p*-values for pairwise baseline–follow-up comparisons.

**Variable**	**Baseline**	**Follow-up**	**P-value**
Immediate recall (REY 1)	6.06 (1.87)	6.81 (2.13)	<0.001
Delayed recall (REY 2)	4.30 (2.14)	4.91 (2.37)	<0.001
Animal fluency (AFT 1)	20.60 (5.55)	20.28 (5.21)	<0.001
Animal fluency (AFT 2)	22.46 (6.32)	22.23 (5.95)	<0.001
Mental alternation (MAT)	27.95 (8.20)	27.34 (7.41)	<0.001
Controlled oral word association (FAS)	39.68 (12.99)	41.08 (12.18)	<0.001
Prospective memory (time-based; TMT)	8.75 (0.77)	8.67 (0.93)	<0.001
Prospective memory (event-based; PMT)	8.61 (1.16)	8.69 (1.07)	<0.001
Stroop (STP)	13.32 (7.35)	13.46 (8.47)	0.026

Descriptive statistics of all cognitive tests under consideration are reported in Table 1 at baseline and follow-up, along with the *p*-values of paired *t*-tests that compared the change in scores over time. A statistically significant (*p*s < 0.05) improvement in scores at follow-up was observed in five out of nine tests, while the remainder of tests (Animal naming, Mental alternation, and the time-based prospective memory test) showed significantly lower (*p* < 0.001) scores at follow-up, compared to baseline. The improvement in scores likely indicates the practice effect of repeated testing and may also partly reflect the continuing strengthening of cognitive skills over time in at least a subset of the participant sample.

#### 2.2.2. Independent Variable

The critical independent variable of this study is perceived loneliness. It is assessed through a single-item question “How often did you feel lonely in the past week?,” which is part of the Center for Epidemiological Studies Short Depression Scale (CESD-10). While four options are available as a response to “How often did you feel lonely?,” due to drastic skewness in the response distribution, we re-coded the responses into a binary variable *LONELY* (see Newall and Menec, 2020) for a similar approach. One level corresponded to option “Never” and accounted for 83% of responses at baseline and another level merged original options “All of the time,” “Occasionally,” and “Some of the time” (17% of responses at baseline). This dichotomization also makes it possible to directly compare results between different studies using the data from CLSA.

We note that the CLSA data contain several measurements pertaining to the individual's social isolation and network, as well as lifestyle and communication practices. Since these measurements tap into constructs that are theoretically and diagnostically distinct from loneliness (Holwerda et al., 2014; Boss et al., 2015; Kuiper et al., 2016), we opted out of combining the loneliness measurement with any of these additional variables into a composite score as this allowed us to focus on the contribution of loneliness to cognitive functioning while maintaining conceptual clarity (Harasemiw et al., 2018; Newall and Menec, 2020). Moreover, this separation provides an opportunity to pinpoint more precisely the potential contribution of loneliness to cognitive functioning. An additional reason for considering a single-item measurement of loneliness stems from our second goal of examining whether this extremely common methodological practice comes with any interpretational or statistical constraints (see section 1).

#### 2.2.3. Covariates

Demographic control variables included age (in years), self-reported sex, rural/urban status (six groups including rural, urban core, and urban fringe) and educational level (coded as 11 levels). Variables related to social network and social support included marital status (single, married or living with a partner, widowed, divorced, separated), number of close friends (outside of family members), and subjective retirement status (retired, semi-retired, not retired). Measures of socio-economic status included total household income in the past 12 months (< $20,000; $20,000 or more but < $50,000; $50,000 or more but < $100,000; $100,000 or more but < $150,000; $150,000 or more) and total value of savings and investments (< $50,000; $50,000 to < $100,000; $100,000 to < $1 million; $1 million or more). Lifestyle variables included type of smoker (six groups ranging from “Daily smoker” to “Never smoked a whole cigarette”) and frequency of alcohol consumption in the past 12 months (eight groups ranging from “6 or more times a week” to “Never”). Most assessments were obtained both at baseline and follow-up, see Table 2. For a detailed description of the variables and their values, readers are referred to the CLSA manual https://www.clsa-elcv.ca/. Jointly, these variables account for major proposed sources of variance in cognitive performance and enable us to determine the unique contribution of loneliness to cognitive functioning and its change due to aging.

**Table 2:** Descriptive statistics of independent variables given as percentages of total sample size, and *p*-values for pairwise baseline–follow-up comparisons for each variable.

**Variable**	**Values**	**Baseline**	**Follow-up**	***P*-value**
Sex				
	Male	47.6	–	
	Female	52.4	–	
Rural/urban status				<0.001
	Group 1 (rural)	8.4	6.5	
	Group 2 (urban core)	86.3	86.2	
	Group 3 (urban fringe)	1.8	2.3	
	Group 4–6 (other)	3.6	4.0	
Education				
	Levels 1–4 (secondary or lower)	12.1	–	
	Levels 5–8 (some post-secondary)	38.5	–	
	Levels 9–11 (university degree)	49.4	–	
Marital status				<0.001
	Single	8.3	8.6	
	Married	73.9	72.8	
	Widowed	6.5	7.4	
	Divorced	9.1	8.8	
	Separated	2.2	2.4	
Number of friends		5.59 (5.69)	5.63 (6.16)	0.435
Loneliness				0.001
(how often feeling lonely)	Never (<1 day/week)	83.4	82.1	
	Occasionally (1 or more days/week)	16.6	17.9	
Retirement status				<0.001
	Retired	39.1	49.5	
	Semi-retired	11.5	10.2	
	Not retired		49.4	40.3
Household income				<0.001
(in thousands CAD)	<20	3.1	2.7	
	20 to <50	17.6	17.0	
	50 to <100	35.6	37.7	
	100 to <150	22.5	21.8	
	>150	21.2	20.8	
Total savings				
(in thousands CAD)	<50	17.9	–	
	50 to <100	14.8	–	
	100 to <1,000	53.5	–	
	>1,000	11.9	–	
Smoking				
	Groups 1–3	7.5	–	
	(daily to occasional smoker)			
	Group 4	39.1	–	
	(former daily smoker, non-smoker now)			
	Group 5	21.9	–	
	(former occasional smoker, non-smoker now)			
	Group 6	31.5	–	
	(never smoked)			
Alcohol consumption				<0.001
	Groups 1–4	63.0	65.3	
	(once a week or more often)			
	Groups 5–8 (never to 2–3 a month)	37.0	34.7	
	(never to 2–3 a month)			

*Percentages for some groups of education level, rural/urban status, type of smoker, and alcohol consumption are reported jointly; analyses are conducted on all groups*.

In addition to estimating the main effects of the critical variable of loneliness and multiple co-variates, we tested select interactions that were identified as theoretically revealing in prior research. Specifically, we considered an interaction between loneliness and educational level in regression models fitted to cognitive test scores, in line with Shankar et al. (2013). Education is a major predictor of an individual's cognitive reserve and has been repeatedly argued to undermine the harmful influence of multiple threats to cognitive functioning, including loneliness (Evans et al., 2018; Farina et al., 2018).

Table 2 reports descriptive statistics of socio-demographic variables (in percentages of the sample size) at baseline and, where available, follow-up. Also reported are *p*-values of pairwise comparisons estimated using the chi-squared test for categorical variables and the paired *t*-test for numeric variables.

### 2.3. Statistical Considerations

CLSA data include sampling weights designed to correct potential biases and deviations of the CLSA sample from the reference population. Thus, all descriptive and inferential analyses in this study incorporate weights by using the packages survey and srvyr in the statistical software platform R version 3.6.3 (R Core Team, 2020). Specifically, survey-weighted generalized linear models were used. We made use of the R packages interactions and jtools for visualization. Finally, all independent variables were treatment coded for the subsequent survey-weighted regression analyses.

We fitted regression models to each of the dependent variables with loneliness as the critical predictor and a number of control variables, listed above, while taking sampling weights into account. We also tested interactions between loneliness and age and loneliness and education. Two sets of models were fitted to analyze (i) the contribution of loneliness at baseline to indices of cognitive functioning at baseline and (ii) the contribution of loneliness at follow-up to the change in cognitive functioning over time, between baseline and follow-up.

To quantify the amount of change in the dependent variable over two testing sessions in (ii), we used residualized change models (Castro-Schilo and Grimm, 2018). In such models, the dependent variable is represented by values of that variable at follow-up, while its values at baseline serve as a predictor. As a result, contributions of all other predictors in the model are estimated against the residual variance in the dependent variable that cannot be explained by its values at baseline. Effectively, these other predictors are tested for whether they explain the change between baseline and follow-up in the dependent variable. For example, scores in the Stroop test measured at follow-up would be modeled as a function of scores in this test measured at baseline, as well as numerous additional predictors, including loneliness, measured at follow-up. An oft-used alternative approach to quantifying the amount of change through difference scores in dependent and critical independent variables was not practical due to a very low reliability of loneliness measurements (see below).

With the present focus on the contribution of loneliness, we only report in the paper those regression models in which the estimate of loneliness reached statistical significance, either as a main effect or in an interaction. However, all regression models are reported in Supplementary Materials (S1). With nine dependent variables at hand (scores in the cognitive tests defined above), a total of 18 regression models were fitted. In each set of nine models, we applied the Bonferroni family-wise correction for multiple comparisons to results of regression models, requiring statistical significance below the 0.006 (0.05/9) level.

## 3. Results

### 3.1. Loneliness as a Predictor of Cognitive Functioning at Baseline

Out of nine models fitted to cognitive test scores, only one—the model fitted to immediate recall scores REY 1 at baseline—has revealed an effect of loneliness at the acceptable Bonferroni-corrected level of statistical significance. Higher levels of education were associated with higher scores in this test across both levels of perceived loneliness. However, immediate recall ability at lower education levels (1–5) was lower in individuals who reported more frequent feelings of loneliness. This finding replicates Shankar et al. (2013) who explain the interaction as a different effect of loneliness on individuals with varying cognitive reserve (see section 4). The results of the survey-weighted regression model is reported as a sequential ANOVA summary table in Table 3 and the corresponding table of coefficients is provided in Supplementary Materials (S1).

**Table 3:** The sequential ANOVA summary table of the survey-weighted regression for REY1 at baseline.

**Variable**	***X*^2^**	***df***	***ddf***	***P*-value**
Marital	216.47	4.00	12002.00	<0.001
Sex	2019.95	1.00	12001.00	<0.001
Age	2595.27	1.00	12000.00	<0.001
Retired	16.71	2.00	11998.00	0.058
Urban/rural	107.45	5.00	11993.00	<0.001
Lonely	15.35	1.00	11992.00	0.049
Education	1620.46	10.00	11982.00	<0.001
Number of friends	19.99	1.00	11981.00	0.02
Smoking	51.94	5.00	11976.00	0.016
Alcohol	546.05	7.00	11807.00	<0.001
Income	99.86	4.00	11803.00	<0.001
Savings	917.97	3.00	11572.00	<0.001
Lonely × Education	77.63	10.00	11562.00	0.002

*df stands for numerator and ddf denominator degrees of freedom*.

Contrary to multiple previous reports (see section 1), we failed to observe a statistically significant effect of perceived loneliness on any other test of memory or executive function in healthy adults at baseline. The results of these models are also reported as sequential ANOVA tables in Supplementary Materials (S1).

**Figure 1:**
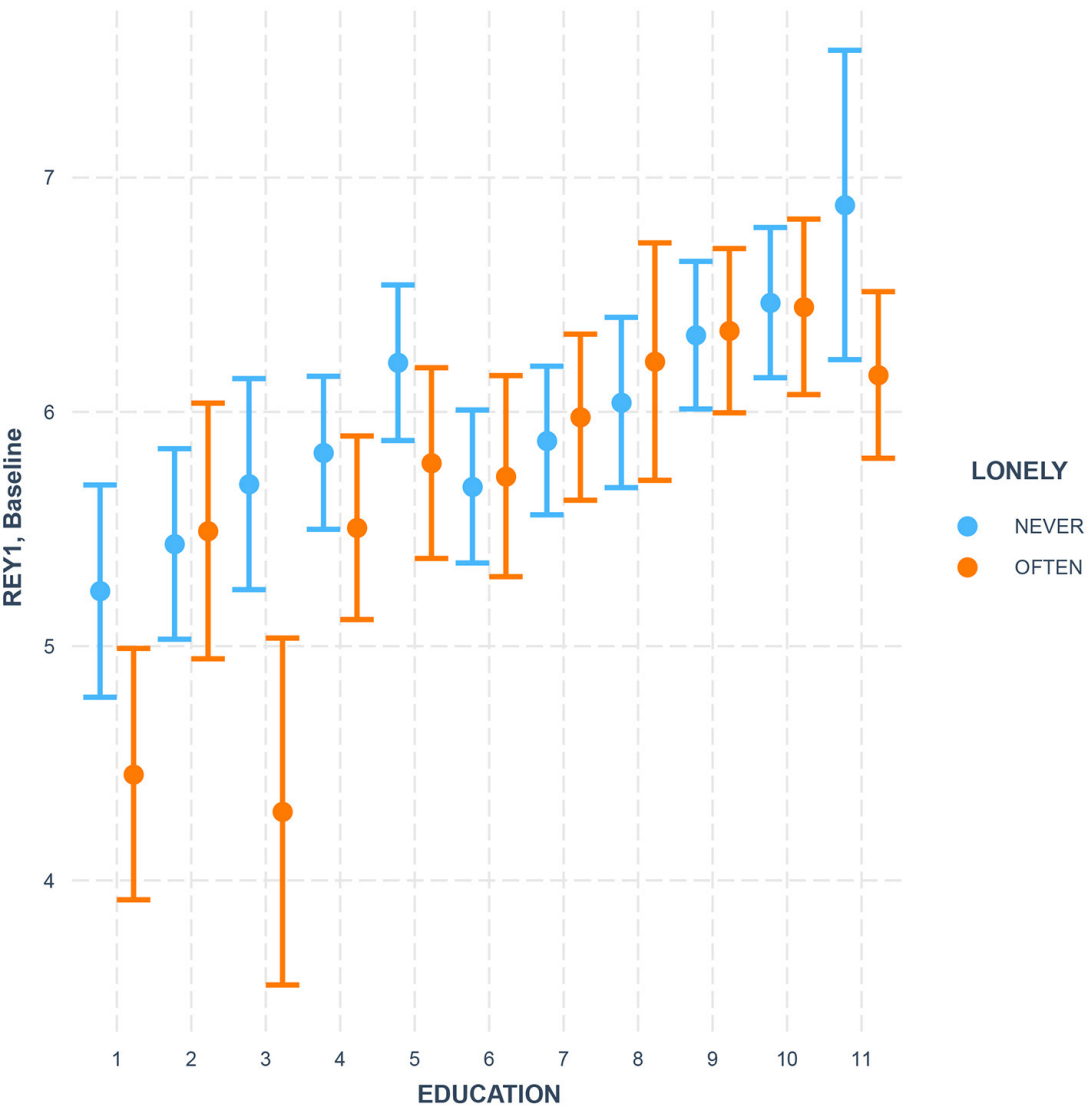
Loneliness × Education interaction as a predictor of immediate recall in the RAVLT memory test (REY 1) at baseline. Education is coded as 11 levels with the following definitions: levels 1–4 (secondary or lower), levels 5–8 (some post-secondary), and levels 9–11 (university degree). Error bars stand for the 95% confidence intervals.

The immediate recall in the RAVLT memory test (*REY 1*) revealed an interaction between loneliness and level of education [*F* = 77.63, *df* = 10, *denom df* = 11,562, *p* = 0.002, see Figure 1]. Higher levels of perceived loneliness were particularly harmful for the immediate recall skill in less-educated groups of participants. This is in line with earlier reports of education as one of the factors that contribute to an individual's cognitive reserve and increase one's resilience to the harmful influence of loneliness and other social factors (Shankar et al., 2013).

### 3.2. Loneliness as a Predictor of Cognitive Change

We estimated changes over time in tests of several cognitive functions by using a series of residualized change regression models (see section 2). Perceived loneliness at follow-up was entered into these regression models as a critical predictor of cognitive change.

The results of the weighted regression model of the time-based test of prospective memory (TMT) at follow-up is provided as a sequential ANOVA summary table in Table 4 and the table of coefficients is provided in Supplementary Materials (S2). Importantly, the estimated effect of loneliness was statistically significant and in the expected direction, namely individuals who reported occasional or frequent feelings of loneliness at follow-up showed a greater loss in the time-based test of prospective memory (TMT) (*b* = −0.078, *t* = −3.255, *p* = 0.001).

**Table 4:** The sequential ANOVA summary table of the survey-weighted regression for TMT at follow-up.

**Variable**	***X*^2^**	***df***	***ddf***	***P*-value**
TMT baseline	415.04	1.00	12221.00	<0.001
Marital	22.50	4.00	12217.00	<0.001
Sex	0.00	1.00	12216.00	0.987
Age	154.08	1.00	12215.00	<0.001
Retired	0.34	2.00	12213.00	0.784
Urban/rural	5.89	5.00	12208.00	0.145
Education	3.12	10.00	12198.00	0.954
Number of friends	0.18	1.00	12197.00	0.578
Smoking	2.99	5.00	12192.00	0.472
Alcohol	17.63	7.00	12185.00	0.002
Income	12.38	4.00	12181.00	0.006
Savings	220.90	3.00	11937.00	<0.001
Lonely	9.32	1.00	11936.00	0.001

The sequential ANOVA summary table of the weighted regression model of the event-based test of prospective memory (PMT) at follow-up is provided in Table 5 and Supplementary Materials (S2) contain the table of coefficients of the model. Similar poorer memory performance was observed with individuals who reported an increased perception of loneliness on the event-based test of prospective memory (PMT) at the follow-up (*b*= −0.068, *t*= −2.994, *p* = 0.003).

**Table 5:** The sequential ANOVA summary table of the survey-weighted regression for PMT at follow-up.

**Variable**	***X*^2^**	***df***	***ddf***	***P*-value**
PMT baseline	532.19	1.00	12280.00	<0.001
Marital	101.64	4.00	12276.00	<0.001
Sex	0.36	1.00	12275.00	0.492
Age	314.19	1.00	12274.00	<0.001
Retired	7.60	2.00	12272.00	0.013
Urban/rural	0.82	5.00	12267.00	0.949
Education	15.74	10.00	12257.00	0.086
Number of friends	3.14	1.00	12256.00	0.224
Smoking	5.98	5.00	12251.00	0.071
Alcohol	9.92	7.00	12244.00	0.069
Income	1.64	4.00	12240.00	0.713
Savings	333.10	3.00	11993.00	<0.001
Lonely	7.15	1.00	11992.00	0.003

After the Bonferroni correction, no other regression model showed either a main or interactive effect of loneliness on any cognitive test score at follow-up. The sequential ANOVA summary tables of these models are provided in Supplementary Materials (S3).

### 3.3. Predictive Validity and Reliability of Loneliness

One of our goals was to establish whether perceived loneliness can be used to predict future cognitive functioning. We pursued this goal in two related ways. First, we examined whether loneliness at baseline was predictive of loneliness at follow-up, within participants. We did so by computing the test-retest reliability of the single-item loneliness measurement in CLSA data. Table 6 reports the distribution of values of *LONELY* over two testing sessions.

**Table 6:** Distribution of perceived loneliness over two testing sessions: unweighted N, weighted N (weighted SE).

	**Follow-up: never**		
	**N (unweighted)**	**N (weighted)**	**SE (weighted)**
Baseline: never	9,042	1,213,514	10,747
Baseline: often	1,077	140,376	5,170
	**Follow-up: often**		
	**N (unweighted)**	**N (weighted)**	**SE (weighted)**
Baseline: never	1,233	160,647	5,550
Baseline: often	968	117,145	4,552

The test-retest reliability kappa over two sessions was very low: κ = 0.34. This aligns well with the fact that the assessment of loneliness used in this study asked how often one felt lonely in the *week* prior to testing, while the interval between the baseline and follow-up testing was 3 years. Since reliability of loneliness measurements over time is low, this puts a substantial constraint on how predictive loneliness at baseline can be for a prognosis of cognitive functioning at a follow-up.

Our second test interrogated predictive validity of baseline loneliness directly. We fitted a series of regression models with cognitive test scores at follow-up as dependent variables and loneliness at baseline as a critical predictor. The remainder of covariates were the same as described in the two sections above. Loneliness at baseline was not a significant predictor of the cognitive performance at follow-up in any of the regression models, after the Bonferroni family-wise correction. We conclude that while loneliness explains variance in some facets of the cognitive ability concurrently (as indicated in the two previous sections), it is not a reliable or valid predictor of the future state of that ability. In section 4, we elaborate on methodological and theoretical implications of this finding.

## 4. General Discussion

There is an apparent consensus in the literature on aging that perceived loneliness correlates with lower levels of cognitive functioning and more rapid cognitive decline in mature adults. As a risk factor to healthy aging, feelings of loneliness have been extensively studied as a predictor of either current or future cognitive impairments (see section 1). Conversely, the effect of loneliness on *healthy* aging has been studied much less, even though the prevalence of perceived loneliness is high in both mid- and late-adulthood (Hawkley and Cacioppo, 2010). Understanding the impact of loneliness on one's cognitive abilities is of paramount importance at the time of the pandemic when large numbers of older adults have been physically or socially isolated from their social supports for extended periods of time. This study harnessed the rich, nationally representative data of the Canadian Longitudinal Study on Aging to examine two questions that have not been resolved in prior research on loneliness in healthy aging. We asked (i) how perceived loneliness affects concurrent cognitive functioning and the change in cognitive functioning over time and (ii) whether perceived loneliness at one point can be used in a prognostic manner, to predict cognitive functioning at a future time-point.

### 4.1. Loneliness Predicts Cognitive Deterioration

We observed that the effect of loneliness on cognitive abilities at baseline was confined to only one test: immediate recall. This negative effect was modulated by education, such that higher incidence of perceived loneliness primarily affected individuals with lower levels of education. This finding replicates results of the English Longitudinal Study of Ageing (Shankar et al., 2013). It also supports the notion that cognitive reserve (of which education is a major predictor) provides compensatory resilience against multiple threats to cognitive functioning, including loneliness (Evans et al., 2018; Farina et al., 2018).

A further analysis of the change in cognitive abilities over time (using residualized scores of CLSA follow-up data) showed that perceived loneliness at follow-up predicted lower levels of time-based and event-based prospective memory, i.e., our capability to remember and execute intentions in the future. The negative impact of loneliness on prospective memory warrants the attention of future studies of cognitive functioning among older adults as the functioning of prospective memory can be linked to successful completion of daily activities in older adulthood (see also Lamichhane et al., 2018). The capability to reliably carry out daily activities is central for maintaining self-sufficiency and allows older individuals to maintain living at home which is connected to increased life satisfaction (Fernández-Portero et al., 2017) and improved mental health status (Muramatsu et al., 2010; Lee et al., 2020).

Taken together, these findings point out that feelings of loneliness correlated with diminished cognitive functioning and a more rapid decrease in functioning over time even in healthy middle-aged and older adults. The magnitude and scope of the loneliness effect on healthy cognitive functioning is more restricted than the prior literature has advocated (Boss et al., 2015; Kuiper et al., 2016). Undoubtedly, a partial explanation of this discrepancy lies with our focus on healthy aging: We removed both clinically depressed individuals and those receiving medical or psychological help at baseline. Another factor may be the precision of measurements and inferential estimates that in our study rely on the superior statistical power and accuracy granted by the CLSA's large sample size, its census-based sampling weights and a comprehensive coverage of cognitive, psychological, social, demographic, and other factors relevant to the study of cognition and aging.

The main contribution of this study is in supplying new longitudinal evidence supporting the view of perceived loneliness as a risk factor for greater and more rapid deterioration of several critical cognitive functions in mid- and late-adulthood. Both the risk of loneliness—a perceived mismatch between one's social needs and affordances—and its harmful effects are exacerbated by the social and physical isolation imposed by the COVID-19 pandemic and related protective measures. As a sizable threat to healthy aging, perceived loneliness is a condition that requires monitoring and intervention in older adults (e.g., Lara et al., 2019). Building up one's cognitive reserve through stimulating mental and social activities and both finding and offering opportunities for individual and group socialization helps “to alleviate the health burden of loneliness” (Hawkley and Cacioppo, 2010, p. 224).

### 4.2. Low Predictive Validity of Loneliness

Findings above confirm the well-described concurrent validity of loneliness: e.g., loneliness at baseline (follow-up) predicts cognitive performance at baseline (follow-up). Yet this study considered a methodological question of practical importance for medical practitioners and caretakers: can measurements of loneliness be used for prognosis of a future change in cognitive functioning in mature adults, i.e., can loneliness at baseline predict cognitive functioning at follow-up. Our consideration was confined to one most common method of operationalizing loneliness, i.e., a single-item question on how often one feels lonely. With minor fluctuations, a single-item question like this is used for measuring loneliness in the majority of cross-sectional and longitudinal studies, including CLSA, as well as standard diagnostic tools of the geriatric mental state.

Perceived loneliness gauged as a single-item question in CLSA showed a low test-retest reliability (κ = 0.34) when comparing the baseline and follow-up responses within participants 3 years apart. This finding has both methodological and theoretical implications. Low reliability suggests the practice of using perceived loneliness measured at a given time-point to predict cognitive functioning at any other time-point to be statistically unwarranted, contra (e.g., Shankar et al., 2013). Furthermore, an individual's level of perceived loneliness at CLSA baseline did not have a significant effect on any of the cognitive functioning test scores at follow-up. Thus, the main contribution of this paper is to demonstrate that the commonly accepted single-item operationalization of loneliness has concurrent validity but not predictive validity. Additional research is required to test whether other, more temporally stable and prognostically predictive operationalizations of loneliness are possible. We speculate that adoption of a more multi-faceted and rigorous measurement of the construct of loneliness in future studies and diagnostic batteries may improve psychometric properties of relevant measures of loneliness and make them viable both for predicting one's concurrent and future mental state.

## Data Availability Statement

The data analyzed in this study is subject to the following licenses/restrictions: data are available from the Canadian Longitudinal Study on Aging (www.clsa-elcv.ca) for researchers who meet the criteria for access to de-identified CLSA data. Requests to access these datasets should be directed to www.clsa-elcv.ca, access@clsa-elcv.ca.

## Ethics Statement

The studies involving human participants were reviewed and approved by Hamilton Integrated Research Ethics Broad (#7783). The patients/participants provided their written informed consent to participate in this study.

## Author Contributions

A-JK and VK contributed equally to the current study and approved the final version. All authors contributed to the article and approved the submitted version.

## Author Disclaimer

The opinions expressed in this manuscript are the authors own and do not reflect the views of the Canadian Longitudinal Study on Aging.

## Conflict of Interest

The authors declare that the research was conducted in the absence of any commercial or financial relationships that could be construed as a potential conflict of interest.

## Publisher's Note

All claims expressed in this article are solely those of the authors and do not necessarily represent those of their affiliated organizations, or those of the publisher, the editors and the reviewers. Any product that may be evaluated in this article, or claim that may be made by its manufacturer, is not guaranteed or endorsed by the publisher.

## References

[B1] Bayard S. Erkes J. Moroni C. (2009). Test du Stroop Victoria-Adaptation Francophone. Collège des Psychologues Cliniciens spécialisés en Neuropsychologie du Languedoc Roussillon (CPCN-LR).

[B2] Bayard S. Erkes J. Moroni C. (2011). Victoria Stroop Test: normative data in a sample group of older people and the study of their clinical applications in the assessment of inhibition in Alzheimer's disease. Arch. Clin. Neuropsychol. 26, 653–661. 10.1093/arclin/acr053 21873625

[B3] Boss L. Kang D.-H. Branson S. (2015). Loneliness and cognitive function in the older adult: a systematic review. Int. Psychogeriatr. 27, 541–553. 10.1017/S1041610214002749 25554219

[B4] Castro-Schilo L. Grimm K. J. (2018). Using residualized change versus difference scores for longitudinal research. J. Soc. Pers. Relationsh. 35, 32–58. 10.1177/0265407517718387

[B5] Conroy R. M. Golden J. Jeffares I. O'Neill D. McGee H. (2010). Boredom-proneness, loneliness, social engagement and depression and their association with cognitive function in older people: a population study. Psychol. Health Med. 15, 463–473. 10.1080/13548506.2010.487103 20677084

[B6] Copeland J. R. M. Dewey M. E. Griffiths-Jones H. M. (1986). A computerized psychiatric diagnostic system and case nomenclature for elderly subjects: GMS and AGECAT. Psychol. Med. 16, 89–99. 10.1017/S0033291700057779 3515380

[B7] de Jong-Gierveld J. Havens B. (2004). Cross-National comparisons of social isolation and loneliness: introduction and overview. Can. J. Aging La Rev. Can. Vieilliss. 23, 109–113. 10.1353/cja.2004.0021 15334811

[B8] Dickens A. P. Richards S. H. Greaves C. J. Campbell J. L. (2011). Interventions targeting social isolation in older people: a systematic review. BMC Publ. Health 11:647. 10.1186/1471-2458-11-647 21843337 PMC3170621

[B9] Ellwardt L. Aartsen M. Deeg D. Steverink N. (2013). Does loneliness mediate the relation between social support and cognitive functioning in later life? Soc. Sci. Med. 98, 116–124. 10.1016/j.socscimed.2013.09.002 24331889

[B10] Evans I. E. M. Llewellyn D. J. Matthews F. E. Woods R. T. Brayne C. Clare L.. (2018). Social isolation, cognitive reserve, and cognition in healthy older people. PLoS ONE 13:e0201008. 10.1371/journal.pone.0201008 30118489 PMC6097646

[B11] Farina M. Paloski L. H. de Oliveira C. R. de Lima Argimon I. I. Irigaray T. Q. (2018). Cognitive reserve in elderly and its connection with cognitive performance: a systematic review. Ageing Int. 43, 496–507. 10.1007/s12126-017-9295-5

[B12] Fernández-Portero C. Alarcón D. Padura Á. B. (2017). Dwelling conditions and life satisfaction of older people through residential satisfaction. J. Environ. Psychol. 49, 1–7. 10.1016/j.jenvp.2016.11.003

[B13] Groarke J. M. Berry E. Graham-Wisener L. McKenna-Plumley P. E. McGlinchey E. Armour C. (2020). Loneliness in the UK during the COVID-19 pandemic: cross-sectional results from the COVID-19 psychological wellbeing study. PLoS ONE 15:e0239698. 10.1371/journal.pone.0239698 32970764 PMC7513993

[B14] Harasemiw O. Newall N. Shooshtari S. Mackenzie C. Menec V. (2018). From social integration to social isolation: the relationship between social network types and perceived availability of social support in a national sample of older Canadians. Res. Aging 40, 715–739. 10.1177/0164027517734587 28982271

[B15] Hawkley L. C. Cacioppo J. T. (2010). Loneliness matters: a theoretical and empirical review of consequences and mechanisms. Ann. Behav. Med. 40, 218–227. 10.1007/s12160-010-9210-8 20652462 PMC3874845

[B16] Heinrich L. M. Gullone E. (2006). The clinical significance of loneliness: a literature review. Clin. Pychol. Rev. 26, 695–718. 10.1016/j.cpr.2006.04.002 16952717

[B17] Holwerda T. J. Deeg D. J. H. Beekman A. T. F. van Tilburg T. G. Stek M. L. Jonker C.. (2014). Feelings of loneliness, but not social isolation, predict dementia onset: results from the Amsterdam Study of the Elderly (AMSTEL). J. Neurol. Neurosurg. Psychiatry 85, 135–142. 10.1136/jnnp-2012-302755 23232034

[B18] Killgore W. D. S. Cloonan S. A. Taylor E. C. Dailey N. S. (2020). Loneliness: a signature mental health concern in the era of COVID-19. Psychiatry Res. 290:113117. 10.1016/j.psychres.2020.113117 32480121 PMC7255345

[B19] Kuiper J. S. Zuidersma M. Zuidema S. U. Burgerhof J. G. M. Stolk R. P. Oude Voshaar R. C.. (2016). Social relationships and cognitive decline: a systematic review and meta-analysis of longitudinal cohort studies. Int. J. Epidemiol. 45, 1169–1206. 10.1093/ije/dyw089 27272181

[B20] Lamichhane B. McDaniel M. A. Waldum E. R. Braver T. S. (2018). Age-related changes in neural mechanisms of prospective memory. Cogn. Affect. Behav. Neurosci. 18, 982–999. 10.3758/s13415-018-0617-1 29926283 PMC6309350

[B21] Lara E. Caballero F. F. Rico-Uribe L. A. Olaya B. Haro J. M. Ayuso-Mateos J. L.. (2019). Are loneliness and social isolation associated with cognitive decline? Int. J. Geriatr. Psychiatry 34, 1613–1622. 10.1002/gps.5174 31304639

[B22] Lee Y. Bierman A. Penning M. (2020). Psychological well-being among informal caregivers in the Canadian longitudinal study on aging: why the location of care Matters. J. Gerontol. Ser. B 75, 2207–2218. 10.1093/geronb/gbaa159 32906145 PMC7664311

[B23] Lezak M. D. Howieson D. B. Loring D. W. Fischer J. S. (2004). Neuropsychological Assessment. Oxford: Oxford University Press.

[B24] Lobo A. LóPez-Antón R. De-La-CÁmara C. Quintanilla M. Á. Campayo A. Saz P. (2008). Non-cognitive psychopathological symptoms associated with incident mild cognitive impairment and dementia, Alzheimer's type. Neurotox. Res. 14, 263–272. 10.1007/BF03033815 19073431

[B25] Loewenstein D. A. Acevedo A. (2004). The Prospective Memory Test: Administration and Scoring Manual. Miami: University of Miami School of Medicine. Unpublished Manuscript.

[B26] Luchetti M. Lee J. H. Aschwanden D. Sesker A. Strickhouser J. E. Terracciano A.. (2020). The trajectory of loneliness in response to COVID-19. Am. Psychol. 75, 897–908. 10.1037/amp0000690 32567879 PMC7890217

[B27] Menec V. H. Newall N. E. Mackenzie C. S. Shooshtari S. Nowicki S. (2019). Examining individual and geographic factors associated with social isolation and loneliness using Canadian Longitudinal Study on Aging (CLSA) data. PLoS ONE 14:e0211143. 10.1371/journal.pone.0211143 30707719 PMC6358157

[B28] Moroni C. Bayard S. (2009). Processus d'inhibition: Quelle est leur évolution après 50 ans? Psychol. NeuroPsychiatr. Vieilliss. 7, 121–129. 10.1684/pnv.2009.0155 19473955

[B29] Muramatsu N. Yin H. Hedeker D. (2010). Functional declines, social support, and mental health in the elderly: does living in a state supportive of home and community-based services make a difference? Soc. Sci. Med. 70, 1050–1058. 10.1016/j.socscimed.2009.12.005 20117865 PMC3360961

[B30] Newall N. E. G. Menec V. H. (2020). A comparison of different definitions of social isolation using Canadian Longitudinal Study on Aging (CLSA) data. Ageing Soc. 40, 2671–2694. 10.1017/S0144686X19000801

[B31] R Core Team (2020). R: A Language and Environment for Statistical Computing. Vienna: R Foundation for Statistical Computing.

[B32] Radloff L. S. (1977). The CES-D scale: a self-report depression scale for research in the general population. Appl. Psychol. Measure. 1, 385–401. 10.1177/01466216770010030623302475

[B33] Raina P. Wolfson C. Kirkland S. Griffith L. E. Balion C. Cossette B.. (2019). Cohort profile: the Canadian longitudinal study on aging (CLSA). Int. J. Epidemiol. 48, 1752–1753. 10.1093/ije/dyz173 31633757 PMC6929533

[B34] Raina P. S. Wolfson C. Kirkland S. A. Griffith L. E. Oremus M. Patterson C.. (2009). The Canadian longitudinal study on aging (CLSA). Can. J. Aging 28, 221–229. 10.1017/S0714980809990055 19860977

[B35] Read D. E. (1987). Neuropsychological assessment of memory in the elderly. Can. J. Exp. Psychol. 41, 158–174. 10.1037/h0084358 3502894

[B36] Rey A. (1964). L'examen clinique en psychologie. [The Clinical Examination in Psychology]. Paris: Presses Universitaries De France.

[B37] Schnittger R. I. B. Wherton J. Prendergast D. Lawlor B. A. (2012). Risk factors and mediating pathways of loneliness and social support in community-dwelling older adults. Aging Ment. Health 16, 335–346. 10.1080/13607863.2011.629092 22129431

[B38] Shankar A. Hamer M. McMunn A. Steptoe A. (2013). Social isolation and loneliness: Relationships with cognitive function during 4 years of follow-up in the English Longitudinal Study of Ageing. Psychos. Med. 75, 161–170. 10.1097/PSY.0b013e31827f09cd 23362501

[B39] Tan J. H. Abdin E. Shahwan S. Zhang Y. Sambasivam R. Vaingankar J. A.. (2019). Happiness and cognitive impairment among older adults: investigating the mediational roles of disability, depression, social contact frequency, and loneliness. Int. J. Environ. Res. Publ. Health 16:4954. 10.3390/ijerph16244954 31817633 PMC6950127

[B40] Teng E. (1995). The mental alternations test (MAT). Clin. Neuropsychol. 9:287.

[B41] Troyer A. K. Leach L. Strauss E. (2006). Aging and response inhibition: aormative data for the victoria stroop test. Aging Neuropsychol. Cogn. 13, 20–35. 10.1080/138255890968187 16766341

[B42] Tuokko H. Griffith L. E. Simard M. Taler V. (2017). Cognitive measures in the Canadian longitudinal study on aging. Clin. Neuropsychol. 31, 233–250. 10.1080/13854046.2016.1254279 27830627

[B43] Wilson R. S. Krueger K. R. Arnold S. E. Schneider J. A. Kelly J. F. Barnes L. L.. (2007). Loneliness and risk of Alzheimer disease. Arch. Gen. Psychiatry 64, 234–240. 10.1001/archpsyc.64.2.234 17283291

