# Supplementary material for: The Effect of Loneliness on Cognitive Functioning Among Healthy Individuals in Mid- and Late-Adulthood: Evidence From the Canadian Longitudinal Study on Aging (CLSA)
Source: Front Psychol. 2021 Sep 3;12:701305. doi: 10.3389/fpsyg.2021.701305 (PMC8448416; doi:10.3389/fpsyg.2021.701305)
Supplement: Supplementary file 1 [file Data_Sheet_1.pdf]

Supplementary Material

SUPPLEMENTARY MATERIALS S1

In this section, we present the summary tables pertaining to the survey-weighted regression models that were fitted on the baseline data. The table of coefficients of the survey-weighted regression model on REY 1 at baseline is given in Table S1. As the categorical independent variables were treatment encoded, the baseline level of a given independent variable is part of the intercept.

Table S1. Table of coefficients of the survey-weighted regression model fitted to immediate recall scores (REY 1) at baseline.

Variable	Est.	*SE*	*t*-value	*p*-value
Intercept	4.668	0.212	22.010	<0.001
Marital_2	0.124	0.069	1.795	0.073
Marital_3	0.113	0.093	1.222	0.222
Marital_4	0.232	0.086	2.677	0.007
Marital_5	0.093	0.131	0.707	0.480
Sex_M	-0.853	0.037	-23.022	<0.001
Age	-0.932	0.048	-19.501	<0.001
Retired_2	0.023	0.057	0.407	0.684
Retired_3	-0.112	0.050	-2.234	0.026
Urban/Rural_1	0.072	0.062	1.159	0.247
Urban/Rural_2	-0.018	0.123	-0.148	0.882
Urban/Rural_4	0.228	0.209	1.089	0.276
Urban/Rural_6	-0.213	0.130	-1.639	0.101
Urban/Rural_9	-0.031	0.213	-0.146	0.884
Lonely_Often	-0.781	0.299	-2.615	0.009
Education_2	0.327	0.200	1.633	0.103
Education_3	0.364	0.224	1.625	0.104
Education_4	0.659	0.168	3.917	<0.001
Education_5	1.028	0.172	5.987	<0.001
Education_6	0.570	0.167	3.405	0.001
Education_7	0.775	0.164	4.717	<0.001

Continued on next page

Table S1 –continued from previous page

Variable	Est.	*SE*	*t*-value	*p*-value
Education_8	0.948	0.182	5.210	<0.001
Education_9	1.212	0.164	7.394	<0.001
Education_10	1.346	0.166	8.120	<0.001
Education_11	1.657	0.300	5.525	<0.001
Number friends	0.057	0.035	1.613	0.107
Smoking_2	0.182	0.163	1.123	0.262
Smoking_3	-0.001	0.315	-0.002	0.998
Smoking_4	0.071	0.077	0.927	0.354
Smoking_5	0.143	0.082	1.754	0.079
Smoking_6	0.211	0.079	2.656	0.008
Alcohol_2	-0.084	0.067	-1.250	0.212
Alcohol_3	-0.143	0.058	-2.457	0.014
Alcohol_4	-0.069	0.068	-1.010	0.312
Alcohol_5	-0.091	0.073	-1.248	0.212
Alcohol_6	-0.158	0.085	-1.875	0.061
Alcohol_7	-0.248	0.077	-3.241	0.001
Alcohol_96	-0.176	0.075	-2.353	0.019
Income_2	0.273	0.119	2.289	0.022
Income_3	0.341	0.120	2.836	0.005
Income_4	0.451	0.127	3.554	<0.001
Income_5	0.479	0.132	3.618	<0.001
Savings_2	-0.134	0.062	-2.167	0.030
Savings_3	0.093	0.053	1.762	0.078
Savings_4	0.070	0.075	0.927	0.354
Lonely_Often:Education_2	0.837	0.407	2.055	0.040
Lonely_Often:Education_3	-0.616	0.489	-1.261	0.207
Lonely_Often:Education_4	0.461	0.331	1.393	0.164
Lonely_Often:Education_5	0.353	0.338	1.042	0.297
Lonely_Often:Education_6	0.825	0.342	2.410	0.016
Lonely_Often:Education_7	0.881	0.316	2.786	0.005
Lonely_Often:Education_8	0.956	0.375	2.551	0.011
Lonely_Often:Education_9	0.800	0.314	2.553	0.011
Lonely_Often:Education_10	0.763	0.317	2.404	0.016
Lonely_Often:Education_11	0.055	0.432	0.128	0.898

Continued on next page

Table S1 – concluded from previous page

Variable	Est.	*SE*	*t*-value	*p*-value

In the remaining survey-weighted regression models, loneliness was not a statistically significant independent variable at baseline. The results of these regression models are presented in Table S2 in the form of an ANOVA summary table.

Table S2. The sequential ANOVA summary table of the survey-weighted regression models at baseline.

Cognitive test	Variable	X^2	*df*	*ddf*	*p*-value
Rey 2	Marital	359.91	4.00	12019.00	<0.001
	Sex	3303.25	1.00	12018.00	<0.001
	Age	3816.60	1.00	12017.00	<0.001
	Retired	28.94	2.00	12015.00	0.027
	Urban/rural	158.05	5.00	12010.00	<0.001
	Education	1533.28	10.00	12000.00	<0.001
	Number of friends	0.56	1.00	11999.00	0.701
	Smoking	48.47	5.00	11994.00	0.071
	Alcohol	805.45	7.00	11825.00	<0.001
	Income	29.58	4.00	11821.00	0.15
	Savings	1124.80	3.00	11588.00	<0.001
	Lonely	0.01	1.00	11587.00	0.96
	Marital	5178.57	4.00	12089.00	<0.001
AFT 1	Sex	183.49	1.00	12088.00	0.032
	Age	31577.89	1.00	12087.00	<0.001
	Retired	341.39	2.00	12085.00	0.003
	Urban/rural	1543.28	5.00	12080.00	<0.001
	Education	16722.07	10.00	12070.00	<0.001
	Number of friends	293.89	1.00	12069.00	0.003
	Smoking	258.48	5.00	12064.00	0.191
	Alcohol	6286.44	7.00	11890.00	<0.001
	Income	828.77	4.00	11886.00	<0.001
	Savings	6373.08	3.00	11651.00	<0.001

Continued on next page

Table S2 –continued from previous page

Cognitive test	Variable	X^2	*df*	*ddf*	*p*-value
	Lonely	10.90	1.00	11650.00	0.566
	Marital	6820.96	4.00	12089.00	<0.001
AFT 2	Sex	603.43	1.00	12088.00	0.001
	Age	39355.20	1.00	12087.00	<0.001
	Retired	464.60	2.00	12085.00	0.003
	Urban/rural	1967.38	5.00	12080.00	<0.001
	Education	23833.82	10.00	12070.00	<0.001
	Number of friends	485.99	1.00	12069.00	0.001
	Smoking	514.60	5.00	12064.00	0.063
	Alcohol	8416.72	7.00	11890.00	<0.001
	Income	1042.74	4.00	11886.00	<0.001
	Savings	9105.25	3.00	11651.00	<0.001
	Lonely	22.01	1.00	11650.00	0.463
	Marital	8421.79	4.00	11873.00	<0.001
MAT	Sex	3991.80	1.00	11872.00	<0.001
	Age	36852.93	1.00	11871.00	<0.001
	Retired	405.34	2.00	11869.00	0.036
	Urban/rural	2358.00	5.00	11864.00	<0.001
	Education	30574.19	10.00	11854.00	<0.001
	Number of friends	469.59	1.00	11853.00	0.012
	Smoking	626.68	5.00	11848.00	0.119
	Alcohol	11616.56	7.00	11684.00	<0.001
	Income	1309.33	4.00	11680.00	0.002
	Savings	15265.17	3.00	11454.00	<0.001
	Lonely	18.27	1.00	11453.00	0.595
	Marital	280.45	4.00	12302.00	<0.001
FAS	Sex	408.72	1.00	12301.00	<0.001
	Age	904.80	1.00	12300.00	<0.001
	Retired	38.70	2.00	12298.00	0.011
	Urban/rural	267.47	5.00	12293.00	<0.001
	Education	3700.62	10.00	12283.00	<0.001
	Number of friends	72.62	1.00	12282.00	<0.001
	Smoking	36.78	5.00	12277.00	0.228
	Alcohol	905.15	7.00	12099.00	<0.001

Continued on next page

Table S2 –continued from previous page

Cognitive test	Variable	X^2	*df*	*ddf*	*p*-value
	Income	63.46	4.00	12095.00	0.006
	Savings	1231.60	3.00	11853.00	<0.001
	Lonely	1.52	1.00	11852.00	0.541
	Marital	4.04	4.00	12218.00	<0.001
TMT	Sex	0.12	1.00	12217.00	0.172
	Age	14.27	1.00	12216.00	<0.001
	Retired	0.34	2.00	12214.00	0.071
	Urban/rural	0.32	5.00	12209.00	0.451
	Education	0.41	10.00	12199.00	0.823
	Number of friends	0.00	1.00	12198.00	0.826
	Smoking	0.55	5.00	12193.00	0.075
	Alcohol	14.29	7.00	12020.00	<0.001
	Income	1.24	4.00	12016.00	0.007
	Savings	20.62	3.00	11776.00	<0.001
	Lonely	0.34	1.00	11775.00	0.037
	Marital	65.89	4.00	12277.00	<0.001
PMT	Sex	2.55	1.00	12276.00	0.133
	Age	596.99	1.00	12275.00	<0.001
	Retired	5.55	2.00	12273.00	0.099
	Urban/rural	6.62	5.00	12268.00	0.073
	Education	47.49	10.00	12258.00	0.001
	Number of friends	2.79	1.00	12257.00	0.221
	Smoking	8.44	5.00	12252.00	0.157
	Alcohol	147.69	7.00	12074.00	<0.001
	Income	11.78	4.00	12070.00	0.035
	Savings	324.81	3.00	11828.00	<0.001
	Lonely	6.98	1.00	11827.00	0.023
	Marital	11051.00	4.00	12302.00	<0.001
	Sex	573.36	1.00	12301.00	0.001
Stroop	Age	89906.24	1.00	12300.00	<0.001
	Retired	498.01	2.00	12298.00	0.004
	Urban/rural	1688.74	5.00	12293.00	0.006
	Education	17735.46	10.00	12283.00	<0.001
	Number of friends	531.47	1.00	12282.00	<0.001

Continued on next page

Table S2 – concluded from previous page

Cognitive test	Variable	X^2	*df*	*ddf*	*p*-value
	Smoking	738.84	5.00	12277.00	0.004
	Alcohol	10904.60	7.00	12099.00	<0.001
	Income	1246.72	4.00	12095.00	<0.001
	Savings	13267.16	3.00	11853.00	<0.001
	Lonely	54.30	1.00	11852.00	0.242

SUPPLEMENTARY MATERIALS S2

In this section, we provide the table of coefficients for those fitted survey-weighted regression models in which loneliness was a statistically significant independent variable at follow-up.

The table of coefficients of the survey-weighted model for the time-based memory test (TMT) is provided in Table S3.

Table S3. Table of coefficients of the survey-weighed regression model for time-based memory test (TMT) at follow-up.

Variable	Est.	*SE*	*t*-value	*p*-value
(Intercept)	8.171	0.244	33.542	0.000
TMT score (baseline)	0.138	0.020	7.010	0.000
Marital_2	-0.004	0.034	-0.127	0.899
Marital_3	0.003	0.053	0.065	0.948
Marital_4	0.018	0.043	0.427	0.669
Marital_5	-0.006	0.062	-0.094	0.925
Sex_M	-0.003	0.016	-0.211	0.833
Age	-0.013	0.002	-8.411	0.000
Retired_2	-0.021	0.029	-0.747	0.455
Retired_3	-0.013	0.023	-0.552	0.581
Urban/Rural_1	0.074	0.037	2.015	0.044
Urban/Rural_2	0.056	0.062	0.902	0.367
Urban/Rural_4	0.104	0.062	1.666	0.096
Urban/Rural_6	0.066	0.059	1.124	0.261
Urban/Rural_9	-0.014	0.075	-0.185	0.853
Education_2	-0.045	0.134	-0.335	0.738
Education_3	-0.073	0.133	-0.545	0.586
Education_4	-0.043	0.109	-0.399	0.690
Education_5	-0.070	0.108	-0.648	0.517
Education_6	-0.063	0.107	-0.584	0.559
Education_7	-0.062	0.107	-0.583	0.560
Education_8	-0.113	0.113	-0.997	0.319
Education_9	-0.108	0.107	-1.010	0.312

Continued on next page

Table S3 – concluded from previous page

Variable	Est.	*SE*	*t*-value	*p*-value
Education_10	-0.129	0.107	-1.208	0.227
Education_11	-0.157	0.346	-0.455	0.649
Number of friends	-0.001	0.001	-1.037	0.300
Smoking_2	0.021	0.057	0.368	0.713
Smoking_3	-0.108	0.134	-0.804	0.421
Smoking_4	0.029	0.035	0.842	0.400
Smoking_5	0.024	0.037	0.655	0.513
Smoking_6	0.049	0.036	1.370	0.171
Alcohol_2	-0.007	0.027	-0.266	0.790
Alcohol_3	-0.018	0.024	-0.771	0.441
Alcohol_4	-0.057	0.030	-1.910	0.056
Alcohol_5	-0.060	0.032	-1.901	0.057
Alcohol_6	-0.043	0.037	-1.177	0.239
Alcohol_7	-0.091	0.034	-2.682	0.007
Alcohol_96	-0.108	0.040	-2.709	0.007
Income2	0.093	0.081	1.147	0.252
Income3	0.164	0.080	2.055	0.040
Income4	0.181	0.082	2.208	0.027
Income5	0.184	0.083	2.215	0.027
Savings2	-0.007	0.028	-0.236	0.813
Savings3	0.014	0.024	0.611	0.541
Savings4	0.048	0.030	1.575	0.115
Lonely_Often	-0.078	0.024	-3.255	0.001

The table of coefficients of the survey-weighted model for the event-based memory test (PMT) is provided in Table S4.

Table S4. Table of coefficients of the survey-weighed regression model for event-based memory test (PMT) at follow-up.

Variable	Est.	*SE*	*t*-value	*p*-value
(Intercept)	8.687	0.247	35.169	0.000
PMT score (baseline)	0.131	0.014	9.528	0.000
Marital_2	0.007	0.031	0.218	0.828
Marital_3	-0.141	0.057	-2.470	0.014
Marital_4	-0.008	0.041	-0.200	0.841
Marital_5	0.000	0.052	0.004	0.997
Sex_M	-0.009	0.016	-0.572	0.567
Age	-0.021	0.002	-12.925	0.000
Retired_2	-0.020	0.033	-0.607	0.544
Retired_3	-0.075	0.027	-2.806	0.005
Urban/Rural_1	-0.000	0.032	-0.010	0.992
Urban/Rural_2	0.027	0.053	0.501	0.617
Urban/Rural_4	0.019	0.074	0.252	0.801
Urban/Rural_6	0.024	0.059	0.406	0.685
Urban/Rural_9	-0.057	0.079	-0.720	0.472
Education_2	0.264	0.194	1.365	0.172
Education_3	0.318	0.195	1.631	0.103
Education_4	0.347	0.172	2.018	0.044
Education_5	0.378	0.172	2.204	0.028
Education_6	0.331	0.171	1.933	0.053
Education_7	0.349	0.170	2.052	0.040
Education_8	0.393	0.173	2.277	0.023
Education_9	0.369	0.170	2.174	0.030
Education_10	0.360	0.170	2.121	0.034
Education_11	0.517	0.180	2.868	0.004
Number of friends	-0.003	0.002	-1.305	0.192

Continued on next page

Table S4 – concluded from previous page

Variable	Est.	*SE*	*t*-value	*p*-value
Smoking_2	0.107	0.049	2.192	0.028
Smoking_3	-0.010	0.092	-0.113	0.910
Smoking_4	0.017	0.035	0.480	0.631
Smoking_5	-0.025	0.037	-0.664	0.507
Smoking_6	0.007	0.036	0.196	0.845
Alcohol_2	-0.020	0.029	-0.709	0.478
Alcohol_3	-0.046	0.024	-1.928	0.054
Alcohol_4	-0.009	0.028	-0.313	0.754
Alcohol_5	-0.056	0.031	-1.782	0.075
Alcohol_6	-0.061	0.038	-1.633	0.102
Alcohol_7	-0.082	0.036	-2.287	0.022
Alcohol_96	-0.068	0.038	-1.821	0.069
Income_2	-0.039	0.070	-0.562	0.574
Income_3	-0.021	0.068	-0.315	0.752
Income_4	-0.016	0.069	-0.231	0.817
Income_5	-0.022	0.071	-0.307	0.759
Savings_2	-0.016	0.029	-0.552	0.581
Savings_3	0.025	0.023	1.056	0.291
Savings_4	0.035	0.033	1.070	0.285
Lonely_often	-0.068	0.023	-2.994	0.003

SUPPLEMENTARY MATERIALS S3

The summary information of the survey-weighted regression models where loneliness was not a statistically significant predictor at follow-up is provided in Table S5 in the form of a sequential ANOVA table.

Table S5. The sequential ANOVA summary tables of the survey-weighted regression models at follow-up.

Cognitive test	Variable	X^2	*df*	*ddf*	*p*-value
REY 1	Baseline score	16513.69	1.00	12005.00	<0.001
	Marital	117.11	4.00	12001.00	<0.001
	Sex	1084.93	1.00	12000.00	<0.001
	Age	1969.19	1.00	11999.00	<0.001
	Retired	39.60	2.00	11997.00	0.003
	Urban/rural	16.52	5.00	11992.00	0.418
	Education	580.33	10.00	11982.00	<0.001
	Number of friends	0.69	1.00	11981.00	0.645
	Smoking	28.83	5.00	11976.00	0.141
	Alcohol	30.79	7.00	11969.00	0.276
	Income	16.50	4.00	11965.00	0.291
	Savings	630.06	3.00	11729.00	<0.001
	Lonely	0.14	1.00	11728.00	0.835
Rey 2	Baseline score	24051.81	1.00	12022.00	<0.001
	Marital	152.39	4.00	12018.00	<0.001
	Sex	1145.57	1.00	12017.00	<0.001
	Age	2508.22	1.00	12016.00	<0.001
	Retired	21.76	2.00	12014.00	0.057
	Urban/rural	20.55	5.00	12009.00	0.463
	Education	748.53	10.00	11999.00	<0.001
	Number of friends	2.19	1.00	11998.00	0.5
	Smoking	52.10	5.00	11993.00	0.029
	Alcohol	33.22	7.00	11986.00	0.345
	Income	13.88	4.00	11982.00	0.464
	Savings	766.69	3.00	11746.00	<0.001

Continued on next page

Table S5 –continued from previous page

Cognitive test	Variable	X^2	*df*	*ddf*	*p*-value
	Lonely	3.26	1.00	11745.00	0.391
AFT 1	Baseline score	125962.95	1.00	12092.00	<0.001
	Marital	1077.95	4.00	12088.00	<0.001
	Sex	3.42	1.00	12087.00	0.687
	Age	6380.64	1.00	12086.00	<0.001
	Retired	113.45	2.00	12084.00	0.054
	Urban/rural	184.16	5.00	12079.00	0.06
	Education	1879.81	10.00	12069.00	<0.001
	Number of friends	4.13	1.00	12068.00	0.761
	Smoking	59.28	5.00	12063.00	0.718
	Alcohol	272.13	7.00	12056.00	0.072
	Income	70.47	4.00	12052.00	0.458
	Savings	4491.49	3.00	11812.00	<0.001
	Lonely	5.40	1.00	11811.00	0.574
AFT 2	Baseline score	176870.19	1.00	12092.00	<0.001
	Marital	1392.85	4.00	12088.00	<0.001
	Sex	150.94	1.00	12087.00	0.02
	Age	7068.81	1.00	12086.00	<0.001
	Retired	176.84	2.00	12084.00	0.025
	Urban/rural	172.07	5.00	12079.00	0.167
	Education	2386.63	10.00	12069.00	<0.001
	Number of friends	2.02	1.00	12068.00	0.844
	Smoking	94.67	5.00	12063.00	0.593
	Alcohol	426.84	7.00	12056.00	0.022
	Income	70.52	4.00	12052.00	0.565
	Savings	5918.15	3.00	11812.00	<0.001
	Lonely	15.00	1.00	11811.00	0.412
MAT	Baseline score	303265.37	1.00	11876.00	<0.001
	Marital	2255.25	4.00	11872.00	<0.001
	Sex	509.60	1.00	11871.00	<0.001
	Age	13291.53	1.00	11870.00	<0.001
	Retired	154.93	2.00	11868.00	0.1
	Urban/rural	138.88	5.00	11863.00	0.58
	Education	3298.22	10.00	11853.00	<0.001

Continued on next page

Table S5 – concluded from previous page

Cognitive test	Variable	X^2	*df*	*ddf*	*p*-value
	Number of friends	116.79	1.00	11852.00	0.068
	Smoking	510.06	5.00	11847.00	0.043
	Alcohol	234.38	7.00	11840.00	0.49
	Income	535.42	4.00	11836.00	0.003
	Savings	6649.45	3.00	11606.00	<0.001
	Lonely	6.57	1.00	11605.00	0.649
FAS	Baseline score	1000602.33	1.00	12305.00	<0.001
	Marital	1162.29	4.00	12301.00	0.001
	Sex	2006.32	1.00	12300.00	<0.001
	Age	4280.27	1.00	12299.00	<0.001
	Retired	288.95	2.00	12297.00	0.145
	Urban/rural	1165.12	5.00	12292.00	0.013
	Education	12594.86	10.00	12282.00	<0.001
	Number of friends	485.79	1.00	12281.00	0.009
	Smoking	803.98	5.00	12276.00	0.074
	Alcohol	1308.14	7.00	12269.00	0.017
	Income	190.31	4.00	12265.00	0.614
	Savings	13878.17	3.00	12018.00	<0.001
	Lonely	135.45	1.00	12017.00	0.162
Stroop	Baseline score	253863.05	1.00	12305.00	<0.001
	Marital	3148.39	4.00	12301.00	<0.001
	Sex	221.70	1.00	12300.00	0.02
	Age	22914.19	1.00	12299.00	<0.001
	Retired	51.49	2.00	12297.00	0.474
	Urban/rural	1182.36	5.00	12292.00	<0.001
	Education	3459.63	10.00	12282.00	<0.001
	Number of friends	27.89	1.00	12281.00	0.386
	Smoking	541.38	5.00	12276.00	0.014
	Alcohol	797.14	7.00	12269.00	0.005
	Income	660.08	4.00	12265.00	0.049
	Savings	17534.82	3.00	12018.00	<0.001
	Lonely	14.40	1.00	12017.00	0.531
